# The Health Food Company

**Published:** 1876-03

**Authors:** 


### *THE HEALTH FOOD COMPANY.*

We are not the especial organ of the Health Food Company, but we rejoice in their success, and are happy to commend to our readers their efforts to supply pure and wholesome foods to the world. We daily experience the advantages which follow the use of genuine articles prepared by this company, and can freely say that we suffer a genuine disappointment whenever the table upon which our food is served lacks the appetizing and nutritious bread made from the Cold Air Attrition Flour, or the delicate, jelly-like mass which the Pearled Wheat affords, or the crisp, parched flavor of the Granulated Wheat Biscuit, or the deep, ruby tint and fruity aroma of the Pomarius. To us and to our family, these and other choice products of the mills and ovens of the Health Food Co. have become a necessity, as we are confident they will to hundreds of thousands of other families, as soon as the merits of these superior foods are widely known. Besides, the Health Food Company are our neighbors, sharing with us the accommodation of our two spacious floors at 137 Eighth Street. This proximity takes us behind the scenes and enables us to speak intelligently of their operations. We can vouch for the care which they exercise in the preparation of all their products, as well as for their promptness and trustworthiness in all business affairs. We are witnesses, also, of much of the great good which they are all the time accomplishing in the building up of broken-down bodies, and the relief of some of the most annoying and painful diseases. Nor is this all. We are in a position to testify to the great moral good accomplished by their efforts. We almost daily hear men and women bearing glad and earnest testimony to the advantages which they have experienced from the exclusive use of these pure, strong, nutritious, simple, and genuine foods, in the place of a stimulating and artifical diet. Some not only admit, but earnestly assert, that the taste for whiskey and tobacco has departed under the benign influence of true foods. Our observation taught us long ago that a properly-fed person, with good digestive powers, has no uncontrollable appetite for alcoholic fluids. It is the uneasy, miserable, sour stomach which craves the biting stimulant or the poisonous sedative; which demands something, it knows not what, and accepts alcohol in some form, because that potent fluid pricks the stomach up to a new sensation while narcotizing the brain and its radiating nerves into a less vivid consciousness of stomachic misery. Seeing all the good results from the very earnest and honest work of our neighbors, why should we not say a good word, as often as we have space, for the Health Food Company? In the interest of our readers and in the advocacy of that "higher life" for humanity, for which we have so long labored, we shall continue to commend to our brother's lips the genuine foods which are here provided for all.

And now let us see what they present that is new, and upon what sort of a basis they found their claims to superior excellence. A few months ago they introduced the preparation which is known as the Cold Air Attrition Whole Wheat Flour. In our last October number we described this flour, and took occasion to object to the name given it. As the process of manufacture is described to us, there is no "attrition" whatever employed. "Attrition" means "to wear with rubbing," and in the new flouring process there is no rubbing at all. The common process of reducing the wheat-berry by grinding between stones could be described by this word more appropriately. The pulverizing power employed by our friends is that of a compressed cold-air blast, which strikes the wheat and dashes it to atoms with tremendous force. We sometime ago suggested that the term "Attrition" be dropped, and that the flour be hereafter known as the

COLD BLAST WHOLE WHEAT FLOUR.

Of this flour we can not speak in too high terms. It is made from choice wheat, which, before pulverizing, is very carefully cleaned. In fact, its outer bark of woody fiber is removed. This is important, as the wheat-berry in its natural state is a rough, scaly structure, bristling with minute hairs and affording abundant hiding-places for the larva of insects. The pulverizing process by the cold-air blast reduces the grains to a fine powder, leaving no bran to be sifted out. Those who eat bread made from this flour get all the nutriment the wheat contains, and that, too, in a form easy of digestion. In the whole wheat meal or flour known as "Graham," even when made from sound wheat, which it is not, as a rule, the bran or hull remains in a coarse, flaky condition, and is not unfrequently a source of active and very unpleasant irritation to the stomach and bowels. Not only this, but the fact that the stomach contracts upon the contents in the natural process of digestion, and that it reluctantly and inadequately contracts when sharp and irritating points present themselves to the delicate lining membrane, shows that minute division of food is necessary to perfect digestion. The woody hull never digests, because it is not a food-substance. It is wise to get it out of our food if we can do so without extirpating some portion of the food substance of the wheat at the same time. By the process of grinding the wheat between stones, it is impossible to retain the most nutritious part of the grain except in the form of hulls. This is because the chief nutriment lies next to the outer woody coat, and is flattened upon it by the grinding act. Sifting through fine silk bolting-cloth separates the white from the dark, the starch from the gluten and hull; that is to say, the white and heating from the dark and strength-giving food. If it were not for the hulls or woody fibers in the bran, it would be a far better food than the white part which is barreled up and sold as flour. But so long as whiteness is considered necessary, so long will our bread be nearly worthless as food. So long as mill-stones are used, so long will the bran contain the best part of the wheat. What suffering humanity needs is a fine flour containing all the food in the wheat. This is found in the Cold-Blast Whole Wheat Flour, beyond a doubt. It is equally certain that those who employ it will get more good from their bread than they have ever before secured. To such, bread will surely be, as never before, "the staff of life."

We have taken a good deal of interest in this flour and the bread and cakes made from it, and have sought information and recipes from all available sources. Some of our friends have succeeded admirably at once in baking this flour, while others have experimented several days before securing perfect results. Miss Julia Coleman, who is known all over the country as a writer upon foods, gives her experience with the Cold-Blast Whole Wheat Flour, and warmly commends its good qualities. She sends the six following recipes for its use:

### PREMIUM BREAD.

Add one part cold, soft water to three parts Attrition Whole Wheat Flour Mix well, and knead a minute or two, or until the mass is homogeneous. Then make into a cylindrical roll, cut off pieces, and roll them with the hand on the board until about three-fourths of an inch thick, and of any convenient length. Put them into a hot oven and bake fifteen or twenty minutes, making them slightly brown. They should be cracked open, light, fine-grained, sweet, and tender. In the course of a few days they become hard, but by returning them to the oven a few minutes they soften and are as good as fresh. If kept dry and clean, they would doubtless be good for months. When wanted to use, dip in cold water before placing in the oven to freshen. They will be found far superior to "Graham" bread.

### ATTRITION WHOLE WHEAT FLOUR GEMS.

To one cup cold, soft water add one and one-fourth cups flour, sifting in the latter slowly while stirring briskly. Make the batter so that it will instantly settle flat after you dip it into the hot gem pans. Bake (but do not scorch) in a very hot oven, for fifteen or twenty minutes. Longer baking makes them tough. They require a hotter oven and less time in it, than "Graham" flour gems.

### SHORT CAKE.

To one cup oatmeal porridge add one cup water, and one and a half cups Attrition Whole Wheat Flour. Beat them well together, and bake in a flat cake, half an inch thick, in a quick oven for fifteen or twenty minutes making it a rich, light brown. Let it stand ten minutes before serving, to avoid its being sticky. If preferred, this batter can be baked in gem pans. The above proportions will need to be varied with the thickness of the porridge or mush, but with a little care it can be made very light and tender.

BERRY SHORT-CAKE.

Make a short-cake like the above. When cool split it open, and lay in the fruit to your taste, return to the oven a few minutes, and then serve.

GRIDDLE CAKES.

Make a batter with equal parts oatmeal porridge, water, and Attrition Whole Wheat Flour. If desired, boiled rice or hominy may be added to it. Bake in a slightly oiled or soapstone griddle, then cover closely, and let them stand a few minutes before serving. Attrition Whole Wheat Flour bakes quicker and browns more nicely than " Graham " flour.

APPLE DUMPLINGS.

Make a batter as above for short-cake. Spread it about half an inch thick in the bottom of the dumpling basin or tins, and place on this cut apples to the depth desired, and cover them with more of the same batter. Then put them into strong steam and cook until the apples are soft. Serve warm with a dressing of Pomarius or sugar to the taste. If preferred, these dumplings can be baked in moderate ovens.

Mr. A. H. Bates, of Orange, Mass, who is using the flour largely, and to whom we submitted Miss Coleman's recipe for Gems, declares that in his experience, perfect success can not be secured unless the batter is made very thin, and is stirred for some minutes. He mixes the Cold-Blast Whole Wheat Flour with very cold-water, stirs five minutes while the pan is heating, then drops a small bit of butter in each pan, stirs the batter for an instant, pours the pans full, and bakes as quickly as possible without burning. He says the gems rise an inch or more, and break open, being light and fine-grained all through. He considers them the best breakfast rolls in the world. He has mailed us some samples which are very fine.

A very palatable and nutritious mush can be made from this Cold-Blast, Whole Wheat Flour, and that with scarcely any labor. We enjoy it very much when cooked at night and allowed to stand till morning, and then served cold with cream and sugar. All that is needed is to wet up the flour with as little cold water as possible, and then stir it, a little at a time, into boiling water, which must be kept over the fire and in violent ebulition, while the flour paste is being added. This destroys the taste of raw wheat as no other process will. Let it boil for fifteen or twenty minutes, and it is done. All kinds of cake, doughnuts, pie-crust—in short, anything which can be made from white flour may be made from this. We ask our lady readers to try it in all possible ways, and to write us the result, telling us at the same time, if they are willing that we should publish such accurate recipes as they may send, and print their names as authority. This will help the good cause, and serve to extend the use of what we are confident is by far the best flour in the world. It sells at $10.00 per barrel, $5.25 per half barrel, and six cents per pound in bags.

Another superior cereal product is the

PEARLED WHEAT.

This, when properly cooked, forms a very handsome dish, as well as a very nutritious and palatable one. It is choice wheat with the rough, outer coat rubbed down so thin that it readily dissolves in water, so that the kernels burst open, and form a homogeneous mass. A double boiler should be used with this as with all wheat foods, so that no stirring is required. Stirring induces a separation of starch and gluten and water, which impairs not only the taste of the food, but its digestibility as well. Without a water-jacketed boiler burning is almost inevitable, in spite of stirring, and carbonized cereals are hard to digest. But if the Pearled Wheat is soaked for six, or eight, or ten hours in cold water, and is then boiled smartly for an hour or two, it will be found simply delicious. We greatly prefer it cold, and find it very agreeable whether used as a vegetable with meats, or as the principal dish for breakfast or supper. It is a very valuable food, and, unlike some good foods, is exceedingly agreeable to every taste. It is far more delicate than crushed or cracked wheat, as may be supposed, and therefore agrees much better with the stomachs of children, invalids and dyspeptics. A very excellent pudding can be made from Pearled Wheat, by stirring into the cold, cooked product a little salt, a pint of milk, a spoonful of sugar, two eggs, and a little grated nutmeg, and baking it. A valuable food for infants is made by stirring the Pearled Wheat into boiling water, adding a trifle of salt, and allowing the mass to boil in a double kettle for an hour or more. When done it must be strained through a thin cloth, so as to exclude all coarse particles. A little sugar may be added, and if the child does not nurse, a little milk. Children thrive wonderfully on this food, and need no other of any name or nature. This supplies all the elements which the human frame contains, and therefore provides for all growth and all waste. Mothers often starve their children by relying upon arrow-root, corn-starch, tapioca and other non-nutritious trash. These are all starchy frauds, disorganizing the digestion, clogging up the secretions, and satisfying the sense of hunger without imparting nourishment. The same is true of all gruels made from white flour. This is about the most wretched diet of all, and the most used. Infant mortality will continue to be enormous until mothers and nurses acquire a little saving knowledge of the chemistry of food. The price of the Pearled Wheat is twenty-five and fifty-five cents per box.

GRANULATED WHEAT (COARSE).

This is choice white wheat, cleaned of its external and obnoxious coat, and then slightly bruised by pounding. It is cooked precisely like the Pearled Wheat, and is greatly superior to all crushed wheats, cracked wheats, and wheaten grits. Its superiority consists in its flavor and in its delicacy, or homogeniety. It reduces readily to a jelly when cooked with abundant water, and will be found the best article of the kind, if we except the Pearled Wheat. Both are greatly superior to rice as food, containing as they do more than double the amount of gluten, and about ten times as much nutritive salts. Both, also, greatly surpass the best oat-preparations, as well as the best hominy or other corn products. We prefer it thoroughly cooked, and thorough cooking to us means long soaking in cold water, and then an hour or more of active boiling in a double boiler. Some tell us that soaking over night and boiling in the morning for twenty or thirty minutes gives them a perfect dish. We have found that this wheat will bear any amount of boiling, if sufficient water is supplied to it, and if all stirring is dispensed with. Even careless handling of the vessel containing the partly cooked wheat will tend to separate the water, and leave it floating upon the top. To agitate the mass in any way is to impair its appearance. The amount of water used should vary according to the amount of boiling. For pearled or granulated wheat which is put in soak at night, and boiled for two hours next day, four parts water to one part wheat is right. But if the soaking process is dispensed with, or the boiling continues but for an hour or less, three parts water will be better than four. The price is $10.50 per bbl., $5.50 per half bbl., and 7 cents per lb. in bags.

GRANULATED WHEAT (FINE).

This is the same choice wheat, well cleaned from its indigestible external coat, and then reduced to a meal by pounding. It is used precisely like "Graham," although it is in every way superior to the best "Graham" to be obtained. This is because it is cleaner, and because pounding is better than grinding for any cereal. Grinding heats the mass, and pounding does not. Heating induces early fermentation. Besides, the rapidly revolving millstone "kills" some portion of every batch ground, and imparts no little grit to the product. These new and better processes of cold-blast pulverising for fine flours, and pounding for the coarser varieties, will prove of vast value in the improvement of our breadstuffs, and we hail their introduction with infinite satisfaction, as will all who have the sense to comprehend the difference. We earnestly commend this fine granulated wheat to those who use "Graham," and who desire to employ only the best. We make superb bread from it by mixing three parts of it to one part of warm water, and baking it in a thin sheet upon the grate of a hot oven. We hardly expect to find a better bread than this. The price of the Granulated Wheat (fine), is the same as that of the Cold-Blast Whole Wheat Flour.

WHITE WHEAT GLUTEN.

Ten thousand mills in the United States are actively engaged in destroying our most valuable cereal—the wheat. The destructive processes are, first, grinding between hot stones, and, second, bolting or sifting out nearly all the food-portion. The Health Food Company now come to the rescue, and start one little mill in just the opposite direction. In place of the grinding, which, let us here remark, they do not employ at all in the preparation of any of their improved foods, they pulverise by the cold-blast and by pounding; instead of robbing the pulverulent mass of its chief food value—its gluten and phosphorous—as do the other ten thousand millers, they eliminate its starch by careful processes, and thus provide a very delicate, gray-colored substance, full of nutriment, and which when boiled and allowed to cool assumes the form of a trembling jelly, easily digested, powerful as a builder-up of weak and delicate persons, and better capable of sustaining the life of the laborer with body or brain, than any other food we have ever seen. It is, in short, a concentrated food, being 58 per cent. gluten, and very rich in phosphatic salts. For nursing mothers and for infants it is a wonderful food. The lacteal secretion is not only increased, but is marvelously enriched by its use. It has still another value, and one of the highest importance to a large class of sufferers. *It has proved itself to possess something like a specific influence over diseases of the kidneys*. A case of diabetes has been greatly modified, and, the patient declares, absolutely cured, under our eye, within the past two months by the use of this White Wheat Gluten to the exclusion of all other food. The case was a severe one, the secretion being nine gallons every twenty-four hours on the average. One of our patients, afflicted with albumenuria, (Bright's disease of the kidneys), told us he had experienced untold comfort from the use of the Gluten. It then occurred to us that the venerable sufferer from diabetes might also find relief by its use. So we ordered it at once, and gave strict injunctions to the patient and attendants that no other food should be eaten. The effect was visible in four hours in the diminished secretion, and has been followed by what seems to be a perfect cure. The theory of this gratifying result is evident, but we need not state it here. We will try to discuss it in a future issue. Dr. Camplin, of London, announced it to the Royal Medical Chirurgical Society as long ago as 1862. He asserted that by freeing the wheat from starch, and retaining only the nitrogen, oil, diastase, and salts, and feeding the patient upon this rich food, the class of diseases of which we speak could be readily controlled. This we will say, here and now, that this White Wheat Gluten is absolutely the best single food we have ever tested, and is well worth a dollar a pound, instead of eight cents. As for the manner of cooking it, perhaps the simplest and best is to make it into mush or porridge. This is done by first wetting it up with a little cold water, afterward stirring it into boiling water until thick enough, and then keeping up the boiling process for twenty minutes. A little salt improves the flavor. Diabetic sufferers must not eat sugar with it; all others can. Cream is always admissable, as is butter or milk. The White Wheat Gluten may be made into bread the same as "Granulated Wheat" (fine) or Cold-Blast. Mixed with eggs, butter, and milk, and baked in thin cakes in a quick oven, it will be found very palatable. An excellent breakfast griddle-cake is made by taking one quart sweet milk, or milk and water, one heaping teaspoonful pure cream-of-tartar mixed dry with the Gluten, or stirred into a stiff batter of Gluten and cold water; one teaspoonful pure bicarbonate of soda dissolved in milk and stirred into the batter, and a little salt. Thin the batter with milk or water so that it will run readily from the spoon. Drop a spoonful of the batter on the griddle, having very slightly greased it first to prevent sticking. If the griddle is of soapstone, no grease will be needed. Let the cakes become brown. Sour milk works splendidly with Gluten, but the cream-of-tartar must be omitted when this is used. Vinegar mixed with water, works well in the absence of cream-of-tartar. Gluten-rice-cakes are made by thoroughly incorporating two parts Gluten with one part nicely cooked rice, and adding one pint or more of sour milk, one egg, one tea-spoonful of soda, and a little salt. Fried to a nice brown, these will be found delicious. Gluten-dice are good eating, and are made as follows: Have the water boiling violently, and keep it in that condition while stirring in Gluten to the consistency of a stiff dough; add a little salt; boil actively for twenty minutes; pour into a dish or pan; when cool and stiff, cut into dice, or slices, and fry in a little lard, browning it all over. This can be eaten with butter or syrup, and may be made up in quantity and fried as demanded. The price of the White Wheat Gluten is $11.00 per barrel, $5.75 per half barrel, and 8 cents per lb. in smaller lots.

THE GRANULATED WHEAT BISCUIT

Are well-known to many of our readers. They are made from Granulated Wheat which has passed through a parching process before being pounded, and are baked in soapstone ovens. They are the only crackers we have ever found which are absolutely perfect in the excellence of the wheat used, in the cleanliness of all the processes, and in the entire absence of grease, soda, baking powders and the like. We have observed that these biscuits are liable to become somewhat tough if exposed to a damp atmosphere. The same is true of all breads made from Whole Wheat Flour. The cure is found in heating them in an oven, or in any way so as to expel the moisture. By this means the food is restored to its original crispness at any time. We can warmly commend these Granulated Wheat Biscuits to all who seek pure, wholesome and nutritious food. The price is 20 cents per lb., or 6 lbs. for $1, and 15 cents per lb. in barrels.

ATTRITION WAFERS.

These are palatable and nutritious, and not objectionable in any way except to such as can not digest food containing sacharine matter. These wafers are thin disks, made from the Cold-Blast Flour, and in order to render them tender and agreeable to children, who frequently do not take kindly at first to plain food, they have a little New Orleans molasses and good butter incorporated in the mixing. They are a very agreeable and useful food, and are of course nutritious, as any food made from this per fect whole wheat flour must needs be. The wafers sell at 25 cents per bl. in any quantity.

POMARIUS.

The uniform testimony is that this is the best fruit product ever made. We know it to be precisely what it purports to be—the juice of sound, ripe, tart, eating apples, reduced to a dense extract by evaporation in porcelain. Many invalids find it very advantageous to use, mixed with water, as a drink, or as a sauce for the Pearled Wheat and other of the farinaceous substances which we have spoken of. We like it at any and all times, and can recommend it with the fullest confidence to sick or well. The price is 50 cents, $1.75, and $3.50.

PEARLED OATS.

This is a preparation of choice oats, from which all hully matter and foreign substances are removed by careful processes. It is used as mush or porridge. We find it to possess a delicate, creamy flavor when boiled for two hours or more, in a double boiler, with four times its bulk of water. It may be cooked in twenty or thirty minutes, if put in a single boiler, in boiling water, and kept violently boiling while being constantly stirred. It makes a very elegant dish by pouring it into a mold and allowing it to cool, and then dropping it into a glass dish. Compared with the best Irish or Scotch or other oat-preparations, it is like a new revelation, being greatly superior to everything of the kind which we have ever tasted. In fact, we doubt if there is anything quite equal to this in the oat line. The price is $11.00 per barrel, $5.75 per half barrel, and 8 cents per pound in bags of various sizes.

DOUBLE BOILERS.

All boiled cereal foods requiring much cooking should be cooked in a double boiler—that is, a boiler having an outer shell containing water, so that the grain shall not come in contact with anything which can burn or char or carbonize it. The juice of carbonized vegetable substances is not objectionable, but in many delicate stomachs the solid parts of articles thus treated are the source of great stomachic uneasiness. It is better always to reject the dish of oatmeal or hominy or wheat or mush which comes to the table possessing the odor or color or flavor of burnt food. The only way to secure the best results with the Pearled Wheat or Granulated Wheat or Pearled Oats is to employ a double boiler. This avoids stirring, and secures the best looking as well as the best flavored dish. The Health Food Company have the best double boilers we have ever seen, both of tin and of enameled iron, holding from a pint and a half to six quarts, and costing from 90 cents to $2.40 each. One or more of these valuable boilers should be in every house, not only for the boiling of cereals, but for milk and other foods.

We are in the secrets of the Health Food Company enough to know that they have some other good things in course of preparation, which will be brought out before long, and that they propose to extend the list of pure and wholesome foods until there shall be no lack of them, either for those who suffer from disease and are seeking health, or for those who are well and prefer to continue so. They have at this time two subjects of importance under consideration—the best food to be secured from choice barley and rye, and the production of a cereal coffee to take the place of coffee made from the coffee-berry, which acts so unfavorably on some constitutions. In all this work we feel a deep interest, as indeed do all who are associated with our JOURNAL OF HEALTH, and are happy to give the benefit of our suggestions and advice, which, we are glad to say, they appear to feel free to ask at all times. We have strongly urged upon them the production of a substitute for coffee which shall possess nutritive properties, and be free from objectionable qualities. A great many persons require a warm drink and feel better for it, especially in the morning. They fly to tea or coffee, and suffer accordingly. The samples of cereal coffee handed us by the Health Food Company are excellent, and make a very palatable and wholesome drink. They have also given us a sample of Granulated Rye, a new preparation made by their pounding process. We find it excellent either for mush or bread, having a delicate, nutty flavor which we have never before found in rye preparations.

We have urged the Health Food Company to keep in stock Packer's excellent Tar Soap and "Charm." It is a convenience to us as well as to others, to have these well-nigh indispensable articles kept at this central point. They therefore offer them for sale at the following rates:

| | | | | | | |
|---|---|---|---|---|---|---|
| For 1 cake Tar Soap or Bottle Charm | | | | | . . . . . . . . . | $0 25 |
| " 3 | " | " | " | " | . . . . . . . . | 65 |
| " 6 | " | " | " | " | . . . . . . . . | 1 25 |
| " 12 | " | " | " | " | . . . . . . . . | 2 40 |

The soap can be sent by mail; the "Charm" can not. When the soap is ordered sent by mail, postage at the rate of seven cents per cake must be added. The Health Food Company ship goods to any point by express; and, if the bill is $5 or more, send C. O. D. if desired. It should be understood, however, that C. O. D. collections are costly arrangements, as a charge is made for the return of the money. In ordering small bills of goods of a responsible house like the Health Food Company, it is better to determine exactly what is wanted and the cost, and remit the amount by draft or post office order, or registered letter. The system of the Health Food Company is to demand a remittance to cover all orders which are to be sent by freight. To the cost of the goods they add from ten to thirty cents for boxes, and twenty-five cents for each heavy package delivered, except at the store, or by hand. Of course express shipments, being taken at the store, cost nothing for delivery.

Every day a number of inquiries reach the Company concerning Agencies for the Health Foods in other cities. To save our friends the trouble of replying to all these in detail, we will say that no such agencies exist, and none are likely to be established. Any dealer can buy the goods, and can make money by keeping them for sale. Friends at a distance who desire to live well, should induce some good grocer to send for a supply of these excellent foods. The address of the Health Food Company is 137 Eighth Street, between Broadway and Fourth Avenue, New York City.

Any person desiring circulars has but to prefer the request on a postal card, and he will receive them by early mail. If samples are desired, from ten to fifty cents in currency will pay for them and for the postage on them. No hesitation need be felt in remitting checks or drafts or postal orders to the Health Food Company, as they are entirely responsible.

---

### *AGENTS.*

**We allow News Dealers, Publishers, and other agents, 60 cents commission for each subscriber obtained at the subscription price—$2. We will make very liberal terms to those who will give their especial attention to procuring subscribers for HALL'S JOURNAL OF HEALTH, the oldest and best health publication in the world.**

